# A Comparative Study of the Epidemiology and Risk Factors of Chronic Kidney Disease Among Rural and Urban Residents in Peshawar, Pakistan

**DOI:** 10.7759/cureus.64215

**Published:** 2024-07-10

**Authors:** Junaid Ali, Shahin Shah, Muhammad Nadeem, Abroo Mahmood, Umair Ahmad

**Affiliations:** 1 General Medicine, Khyber Medical University, Peshawar, PAK; 2 General Medicine, Medlife Medical Center, Abu Dhabi, ARE; 3 International Fellow Training, University Hospitals Birmingham NHS Foundation Trust, Birmingham, GBR; 4 General Medicine, Advocare Northbrunswick Medical Associates, Abu Dhabi, ARE; 5 Medicine, Khyber Pakhtunkhwa Health Department, Peshawar, PAK

**Keywords:** glomeruler disease, estimated glomerular filtration rate (egfr), albuminuria, obstructive nephropathy, chronic kidney disease

## Abstract

Background and objective

Chronic kidney disease (CKD) poses a significant global public health challenge, especially among the Asian population who experience higher prevalence and more rapid disease progression. This study aimed to compare the epidemiology and risk factors associated with CKD between rural and urban residents in Peshawar, Pakistan.

Materials and methods

A cross-sectional study involving adult patients with CKD was conducted at a public tertiary care hospital in Peshawar between July 2023 and January 2024. To collect data, a tool was developed based on existing literature. CKD was defined as follows: a low estimated glomerular filtration rate (eGFR) below 60 mL/min per 1.73 m^2^, albuminuria (urine albumin-creatinine ratio >3 mg/mmol), or a combination of both low eGFR and albuminuria. The prevalence of moderate to severe CKD, adjusted for place of residence, was calculated. Statistical analysis was performed using SPSS Statistics V. 26 (IBM Corp., Armonk, NY).

Results

Among the study sample, 114 (41.45%) patients hailed from rural areas while 161 (58.55%) resided in urban areas. Urban patients had a higher prevalence of albuminuria levels below 30 mg/g than rural patients (83.2% vs. 76.3%, p=0.00). Additionally, the mean eGFR was slightly higher among rural residents. Rural patients had a higher prevalence of hypertension, and there was a noticeable disparity in the occurrence of kidney stones, with rural residents experiencing a greater incidence. Patients living in urban areas showed a higher level of understanding of risk factors and reported taking preventive measures for CKD. Factors associated with moderate to severe CKD included living in urban areas and having a medical history of diabetes and hypertension (p=0.00). No significant association was observed between behavioral factors and the severity of CKD.

Conclusions

Urban residents exhibited higher rates of CKD and albuminuria and had a greater awareness of CKD risk factors. In contrast, rural areas had a slightly higher mean eGFR and greater prevalence of hypertension and kidney stones. Diabetes and hypertension were key predictors of moderate to severe CKD.

## Introduction

Chronic kidney disease (CKD) is characterized by a gradual decline in renal function that can persist for an extended period. Injuries to the kidneys can arise from physical trauma, diabetes mellitus (DM), or hypertension. Damaged kidneys are unable to filter blood and perform other essential functions. Proteinuria and a decrease in glomerular filtration rate (GFR) are frequently observed indicators of this condition [1]. CKD has emerged as a major cause of illness and death in the modern era, impacting a substantial portion of the world's population [2]. There has been a noticeable increase in the prevalence of CKD due to the rise in various risk factors such as obesity and DM [3]. Although mortality rates have decreased for patients with end-stage kidney disease (ESKD), the Global Burden of Disease (GBD) study reveals that CKD has emerged as the leading cause of death worldwide [4]. Based on an extensive analysis of 100 studies with over 6.9 million patients, the global prevalence of CKD stages 1-5 was 13.4%, with stages 3-5 accounting for 10.6% [5].

The literature examines a multitude of risk factors linked to chronic renal disease. Hypertension and DM are the primary causes of CKD in around 66% of individuals affected by the condition globally [6]. In addition, CKD can be exacerbated by some detrimental lifestyle factors, including smoking, excessive alcohol consumption, and insufficient nutrition [7]. In rural areas, the primary and significant risk factors involve environmental pollution, the use of contaminated water, and lack of adequate healthcare. Concurrently, individuals residing in metropolitan areas may face significant risk factors due to their proximity/access to contaminated industrial goods and the adoption of a sedentary way of life [8,9]. Literature has focused on investigating the risk factors of CKD in various regions of South Asia and observed that multiple factors can trigger the development of the disease [10].

The combination of genetic susceptibility, low standard of living, poor economic status, particular cultural practices, and restricted availability of public welfare services has been implicated in the spread of CKD. A local study conducted in Pakistan highlighted the limited awareness among rural inhabitants about CKD and its associated risk factors [11]. Rural residents' reluctance toward timely medical treatment is influenced by their preference for traditional medicines and limited access to the formal schooling system. Simultaneously, the urban population may encounter the challenge of persistent stress and heightened susceptibility to lifestyle disorders [12]. In light of the aforementioned variables, the objective of the study is to examine the epidemiology and risk factors linked to CKD in both rural and urban inhabitants of Peshawar, Pakistan. The study aims to identify significant sociodemographic, clinical, and behavioral disparities between these groups, which can enhance our understanding of the prevalence of and various factors influencing CKD.

## Materials and methods

This study was conducted at a public tertiary care hospital in Peshawar, Pakistan, from July 2023 to Jan 2024. The study population comprised all adult patients irrespective of gender who were attending the medicine and nephrology outpatient department of the hospital. Patients with acute kidney disease, those with incomplete medical records, or those not willing to give consent were excluded from the study. The sample size needed for the study was calculated to be 275. The sample estimation was based on the CKD prevalence of 23.3% reported by The Pakistan Demographic Health Survey (PDHS) 2017-2018 [13]. By factoring in a 10% assumed non-response rate, we approached 303 potential participants and invited them to participate; 282 participants filled out the survey questionnaire. Seven of the participants’ responses were incomplete and hence excluded from the final analysis.

The data collection tool used for this study was designed based on an extensive literature review to make the data consistent and relevant. The data collected were classified into sociodemographic, clinical, behavioral, and anthropometric data. The sociodemographic and behavioral data were collected from patients or their attendants while anthropometric measurements and hypertension status were taken by the nurses on duty. Laboratory findings such as diabetes status, estimated GFR (eGFR), Aabuminuria, and provisional diagnosis data were obtained from the patient's medical files.

The primary focus of this research was CKD, which was defined as the presence of albuminuria (albumin/creatinine ratio ≤30 mg/g) for at least three months and/or a decreased eGFR (<60 ml/min/1.73 m^2^) as per the Kidney Disease: Improving Global Outcomes (KDIGO) guidelines [14]. The exposure variables comprised demographic information (age and gender), self-reported pre-existing conditions (hypertension, diabetes, and gout), and lifestyle choices (smoking, physical activity, and diet). Additionally, the use of nephrotoxins, energy drinks, and herbal medicines, CKD awareness, and health education were also assessed. The data was collected verbally by the research assistants in the Pashtu language. The participants were informed about the objectives of the study after obtaining verbal agreement. This study proposal was approved by the Research and Ethics Committee of the Institute of Public Health Peshawar, Pakistan (Re: IPH&SS-AAA-325).

The data were analyzed using SPSS Statistics V. 26 (IBM Corp., Armonk, NY). A comprehensive analysis, including both descriptive and analytical approaches, was conducted for all variables. The data were presented as the mean value plus or minus the standard deviation (SD), or as frequencies and percentages, as deemed appropriate. Following the assessment of data normality, a comparison was performed using suitable statistical tests, such as the chi-square test and t-test. Residence-adjusted logistic regression was performed for the assessment of risk factors of moderate to severe CKD. A p-value <0.05 was considered statistically significant.

## Results

A total of 275 CKD patients were included in the final analysis. The mean age of the sample was 48 ± 112 years. Among them, 114 (41.45%) patients resided in adjacent rural areas of the province, while 161 (58.55%) belonged to urban areas. In terms of gender, 202 (73.5%) were male, and 73 (26.5%) were female. Regarding age distribution, 55 (20.0%) were below 40 years, 124 (45.1%) were between 41 and 50 years, and 96 (34.9%) were above 50 years. The sociodemographic characteristics of the study sample are presented in Table 1.

**Table 1:** Comparison of sociodemographic characteristics between urban and rural patients ^*^P<0.05 (chi-square test) CKD: chronic kidney disease

Variables	Overall, n (%)	Urban, n (%)	Rural, n (%)	P-value
Gender				0.00^*^
Male	202 (73.5)	106 (65.8)	96 (84.2)	
Female	73 (26.5)	55 (34.2)	18 (15.8)	
Age, years				0.01^*^
<40	55 (20.0%)	25 (15.5%)	30 (26.3%)	
41-50	124 (45.1%)	65 (40.4%)	59 (51.8%)	
>50	96 (34.9%)	71 (44.1%)	25 (21.9%)	
Marital status				0.31
Married	254 (92.4%)	149 (92.5%)	105 (92.1%)	
Unmarried	10 (3.6%)	6 (3.7%)	4 (3.5%)	
Divorced	8 (2.9%)	3 (1.9%)	5 (4.4%)	
Widowed	3 (1.1%)	3 (1.9%)	0	
Highest educational qualification				0.06
Primary	62 (22.5%)	38 (23.6%)	24 (21.1%)	
Higher secondary school	155 (56.4%)	81 (50.3%)	74 (64.9%)	
Intermediate	45 (16.4%)	32 (19.9%)	13 (11.4%)	
Bachelor and above	13 (4.7%)	10 (6.2%)	3 (2.6%)	
Monthly income				0.03^*^
Less than 15K	101 (36.7%)	57 (35.4%)	44 (38.6%)	
16-30K	134 (48.7%)	88 (54.7%)	46 (40.4%)	
Above 30K	40 (14.5%)	16 (9.9%)	24 (21.1%)	
Housing				0.46
Mud	91 (32.6%)	57 (35.4%)	34 (29.8%)	
Cemented	147 (52.7%)	81 (50.3%)	66 (57.9%)	
Tiled	37 (13.5%)	23 (14.3%)	14 (12.3%)	
Occupational status				0.43
Labor	138 (50.2%)	86 (53.4%)	52 (45.6%)	
Hawker	61 (22.2%)	34 (21.1%)	27 (23.7%)	
Professional	76 (27.6%)	41 (25.5%)	35 (30.7%)	
Family history of CKD				0.20
Yes	97 (35.3%)	62 (38.5%)	35 (30.7%)	
No	178 (64.7%)	99 (61.5%)	79 (69.3%)	

**Figure 1:**
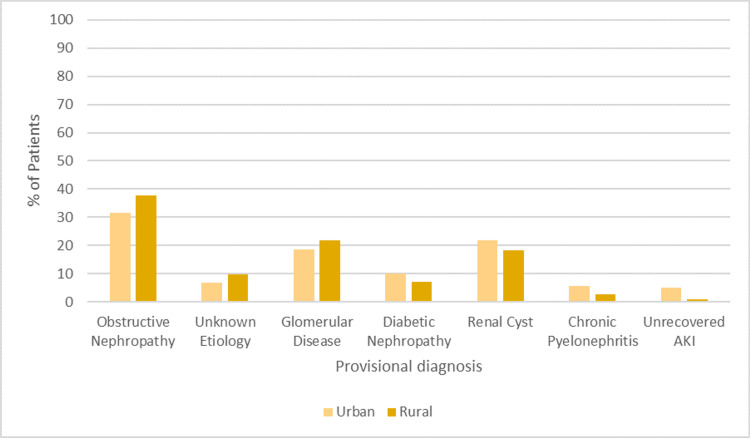
Comparison of provisional diagnosis between two groups AKI: acute kidney injury

Figure 1 depicts the provisional diagnoses among patients, with a total of 275 participants, including 161 (58.5%) urban and 114 (41.5%) rural residents. The most common provisional diagnosis was obstructive nephropathy, accounting for 94 (34.2%) cases overall, with a slightly higher prevalence in rural areas (n=43, 37.7%) compared to urban (n=51, 31.7%), although this difference was not statistically significant (p=0.491). Cases with unknown etiology constituted 8.0% (n=22) of diagnoses, with a similar distribution between urban (n=11, 6.8%) and rural (n=11, 9.6%) populations.

**Figure 2:**
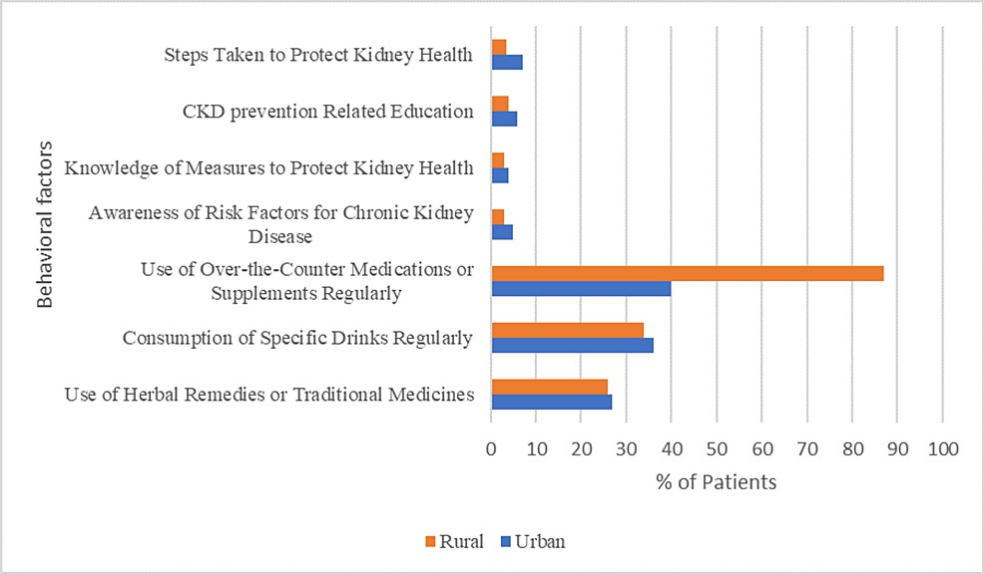
Behavioral factors in the study sample regarding CKD prevention CKD: chronic kidney disease

Table 2 and Figure 2 depict the clinical and behavioral factors associated with CKD among the study sample. A significant difference was observed in the prevalence of hypertension (p=0.04); 117 (42.5%) participants reported hypertension, with a higher prevalence in rural residents (n=50, 43.9%) compared to urban (n=67, 41.6%). Conversely, the presence of kidney stones (p=0.00) showed a marked disparity, as 86 (62.9%) of individuals with a history of kidney stones were rural residents compared to 87 (54.0%) urban. Physical activity levels displayed a notable trend (p=0.02), with 142 (51.8%) of participants engaging in active physical activities, slightly higher in urban areas (n=85, 52.8%) than in rural (n=57, 50.0%). Other variables like diabetes, heart condition, BMI intervals, dietary habits, and blood pressure did not exhibit statistically significant differences between urban and rural populations.

**Table 2:** Clinical and behavioral factors among the study sample ^*^P<0.05 (chi-square test) BMI: body mass index

Variables	Overall, n (%)	Urban, n (%)	Rural, n (%)	P-value
History of kidney stones				0.00^*^
Yes	173 (62.9)	87 (54.0)	86 (75.4)	
No	102 (37.1)	74 (46.0)	28 (24.6)	
Diabetes				0.32
Yes	160 (58.2)	98 (60.9)	62 (54.4)	
No	115 (41.8)	63 (39.1)	52 (45.6)	
Hypertension				0.04^*^
Yes	117 (42.5)	67 (41.6)	50 (43.9)	
No	158 (57.5)	94 (58.4)	64 (56.1)	
Heart condition				0.71
Yes	104 (37.8)	59 (36.6)	45 (39.5)	
No	171 (62.2)	102 (63.4)	69 (60.5)	
BMI intervals				0.74
Underweight	23 (8.4)	11 (6.8)	12 (10.5)	
Normal BMI	124 (45.1)	73 (45.3)	51 (44.7)	
Overweight	114 (41.5)	69 (42.9)	45 (39.5)	
Obese	14 (5.1)	8 (5.0)	6 (5.3)	
Dietary habits				0.57
Mostly vegetarian	78 (28.4)	49 (30.4)	29 (25.4)	
Mostly non-vegetarian	118 (42.9)	69 (42.9)	49 (43.0)	
Mixed	79 (28.7)	43 (26.7)	36 (31.6)	
Blood pressure				0.14
Not controlled	88 (31.9)	46 (28.6)	42 (36.8)	
Controlled	187 (68.1)	115 (71.4)	72 (63.2)	
Physical activities				0.02^*^
Sedentary	48 (17.4)	31 (19.3)	17 (14.9)	
Moderate	85 (30.8)	45 (28.0)	40 (35.1)	
Active	142 (51.8)	85 (52.8)	57 (50.0)	

In urban areas, 44 (27.3%) participants used herbal remedies, 59 (36.7%) consumed specific drinks (energy and herbal drinks) regularly, and 64 (39.8%) used over-the-counter medications. Awareness of CKD risk factors was noted in eight (5.0%), knowledge of kidney protection measures in seven (4.3%), CKD prevention awareness in nine (5.6%), and 12 (7.5%) took steps to protect kidney health. In rural areas, 30 (26.3%) used herbal remedies, 39 (34.2%) consumed specific drinks regularly, and 99 (86.8%) used over-the-counter medications. Awareness of CKD risk factors was found in four (3.5%), knowledge of kidney protection measures in three (2.6%), CKD prevention awareness in four (3.5%), and four (3.5%) took steps to protect kidney health.

The mean serum creatinine levels were similar between rural and urban participants (10.6 ± 1.5 mg/l overall, p=0.319). Regarding albuminuria, a significant difference was found between rural and urban populations (p=0.007), with 87 (76.3%) rural participants and 134 (83.2%) urban participants having albuminuria levels <30 mg/g. Moderate albuminuria (30-300 mg/g) was present in 20 (17.5%) rural participants compared to 18 (11.2%) urban participants. The mean eGFR was slightly higher in rural areas compared to urban areas across different equations, with Modification of Diet in Renal Disease (MDRD) showing 109.5 ± 15.0 ml/min overall (p=0.12). In terms of kidney function stages using the MDRD equation, 88 (77.2%) rural participants and 104 (64.6%) urban participants had eGFR >90 ml/min/1.73 m² (p=0.04), while the Chronic Kidney Disease Epidemiology Collaboration (CKD-EPI) equation showed similar trends with 91 (79.8%) rural participants and 103 (64.0%) urban participants having eGFR >90 ml/min/1.73 m² (Table 3).

**Table 3:** Kidney function test and urine profile ^*^P<0.05 (independent t-test) CKD-EPI: Chronic Kidney Disease Epidemiology Collaboration; eGFR: estimated glomerular filtration rate; MDRD=Modification of Diet in Renal Disease; SD: standard deviation

Characteristics	Overall (n=275)	Rural (n=114)	Urban (n=161)	P-value
Mean serum creatinine, mg/l, mean ± SD	10.6 ± 1.5	10.3 ± 2.1	11.6 ± 3.0	0.31
Albuminuria, mg/g, n (%)				0.00^*^
<30	221 (80.4)	87 (76.3)	134 (83.2)	
30-300	38 (13.8)	20 (17.5)	18 (11.2)	
>300	16 (5.8)	7 (6.1)	9 (5.6)	
Mean eGFR, ml/min, mean ± SD				0.12
MDRD	109.5 ± 15.0	110.9 ± 16.2	105.8 ± 18.4	
CKD-EPI	106.4 ± 13.5	107.2 ± 14.1	104.2 ± 15.3	
Stages of kidney function by eGFR, n (%)				0.04^*^
MDRD, ml/min/1.73 m², n (%)				
>90	192 (69.8)	88 (77.2)	104 (64.6)	
60-90	56 (20.4)	16 (14.0)	40 (24.8)	
<60	27 (9.8)	10 (8.8)	17 (10.)	
CKD-EPI, ml/min/1.73 m², n (%)				
>90	194 (70.5%)	91 (79.8)	103 (64.0)	
60-90	51 (18.5)	14 (12.3)	37 (23.0)	
<60	30 (10.9)	9 (7.9)	21 (13.0)	

In the residence-adjusted logistic regression analysis as shown in Table 4, significant predictors of moderate to severe CKD stages G3-G4 were identified. Living in a rural area was associated with significantly lower odds of moderate to severe CKD (OR: 0.18, 95% CI: 0.05-0.75, p=0.01). A history of diabetes was a strong predictor, with individuals with diabetes having over four times the odds of developing moderate to severe CKD compared to those without diabetes (OR: 4.35, 95% CI: 1.50-12.65, p=0.00). A history of hypertension showed higher odds (OR: 4.60, 95% CI: 0.82-25.88, p=0.00), demonstrating a significant association with CKD severity. Other factors, including smoking status, use of herbal medicines, energy drink consumption, higher BMI, and sedentary physical activity, were not significantly associated with moderate to severe CKD stages.

**Table 4:** Residence-adjusted predictors of moderate to severe CKD in the study sample Regression analysis. ^*^P<0.05 BMI: body mass index; CI: confidence interval; CKD: chronic kidney disease; OR: odds ratio

Variables	CKD stages G3-G4, OR (95% CI)	P-value
Rural residence	0.18 (0.05-0.75)	0.01^*^
Family history of CKD disease		
History of hypertension	4.60 (0.82-25.88)	0.00^*^
History of diabetes	4.35 (1.50-12.65)	0.00^*^
History of heart diseases		
Smoking status	0.15 (0.07-10.29)	0.17
Use of herbal medicines	0.30 (0.06-1.35)	0.12
Use of pain medications		
Consumption of energy drinks	1.01 (0.98-1.04)	0.15
BMI ≥25 kg/m^2^	1.01 (0.96-1.07)	0.32
Sedentary lifestyle	2.75 (0.63-12.02)	0.16

## Discussion

The results of the current study indicate that the prevalence of CKD among rural residents was relatively low as compared to urban residents. Risk factors such as a history of diabetes and hypertension were also more prevalent among urban residents. The research revealed that obstructive nephropathy is the most common cause of CKD, which aligns with the results of a prior local study [15]. Recent urban research has shown that chronic glomerulonephritis and diabetic nephropathy (DNP) are the primary causes of moderate to severe CKD, surpassing kidney stones in prevalence. Diabetes has been the primary cause of CKD in recent times, perhaps due to the massive urbanization that has occurred in Pakistan during the last several decades [15,16].

The current analysis demonstrated rural residence to be a protective factor. Our finding showed that residing in a rural area significantly reduces the odds of developing moderate to severe CKD (OR: 0.18, 95% CI: 0.05-0.75, p=0.01), which aligns with previous research suggesting that rural living is associated with potential protective effects against the development of CKD. This might be attributed to differences in lifestyle, dietary factors, and environmental exposures between rural and urban settings. For example, rural populations may have less exposure to some urban pollutants and stressors that contribute to CKD progression [17,18,19].

Our results are consistent with previous research and showed a significant relationship between diabetes and hypertension with CKD [20,21]. Otherwise, the focus should necessarily be on the effective treatment of diabetes as 4.6 times higher odds for moderate to severe CKD (OR: 4.35, 95% CI: 1.50-12.65, p=0.00) was observed in diabetics compared to non-diabetics. This strong inter-relationship between diabetes and kidney disease highlights the need to screen for renal function in the diabetic population for early management of CKD. Good glycemic and blood pressure control and treatment of diabetic kidney diseases aim at the best possible communication between different specialties and preventing this entity by using medications and healthy lifestyle modifications, among patients with DM-related CKD [21]. Early detection and management are crucial for positive clinical outcomes of CKD in diabetic patients [22].

The disparities between our study and existing literature underscore the complexity of CKD risk factors. While our findings did not show significant associations between CKD and factors such as smoking, herbal medicine use, and sedentary lifestyle, other studies have reported strong links. For instance, Fadlilah et al. found significant associations with age, smoking behavior, diabetes mellitus, and hypertension [23], while Wang et al. highlighted the impact of lifestyle risk factors on CKD development [24]. Cha and Han also emphasized the role of diabetes, hypertension, and smoking in CKD progression [25]. These differences suggest a need for further research to understand the varying impact of risk factors across different populations. These disparities also highlight the intricate correlation between risk factors for CKD. In our cohort, it seems that the impact of factors such as obesity and physical inactivity may be less significant or overridden by more influential predictors such as diabetes and residing in rural areas. Additional investigation is required to provide a more comprehensive understanding of the complex interrelationships among different elements in diverse populations.

This study has certain limitations, including its small sample size and the fact that the sample may not be representative of all rural and urban populations in Peshawar, limiting the generalizability of the findings. Studies involving larger, more diverse cohorts would provide more robust data. Secondly, the cross-sectional study design did not allow for the establishment of causality between risk factors and CKD. Longitudinal studies are recommended to better understand the temporal relationships between these factors and CKD progression. Additionally, the reliance on self-reported data is also a limitation of the current study, as it may have introduced reporting bias.

## Conclusions

Urban inhabitants are likely to have a higher prevalence of CKD than rural residents. Patients living in urban areas had a higher likelihood of having albuminuria levels below 30 mg/g, although rural people had a slightly higher average eGFR. On the other hand, rural patients had a higher prevalence of hypertension. The prevalence of kidney stone history was markedly higher among rural residents compared to other groups. Patients residing in urban areas also demonstrated elevated levels of awareness of risk factors and preventative measures for CKD. The risk variables associated with moderate to severe CKD include urban residency, a history of diabetes, and hypertension.
